# Structure–Function Relationship Studies of Multidomain Levansucrases from Leuconostocaceae Family

**DOI:** 10.3390/microorganisms10050889

**Published:** 2022-04-24

**Authors:** Flor de María García-Paz, Salvador Martínez-Bahena, Clarita Olvera

**Affiliations:** Departamento de Ingeniería Celular y Biocatálisis, Instituto de Biotecnología, Universidad Nacional Autónoma de México, Avenida Universidad 2001 Col. Chamilpa, Cuernavaca CP 62210, Mexico; flor.garcia@ibt.unam.mx (F.d.M.G.-P.); salvador.martinez@ibt.unam.mx (S.M.-B.)

**Keywords:** multidomain fructansucrases, levan, levansucrase, enzyme structure/function relationship, polymers

## Abstract

Levansucrase LevS from Leuconostoc mesenteroides B-512F is a multidomain fructansucrase (MD-FN) that contains additional domains (ADs) to the catalytic domain. However, the understanding of the effect that these ADs have on enzyme activity remains vague. To this aim, structure-function relationship studies of these LevS ADs were performed by evaluating both biochemical properties and the enzymatic capacity of truncated versions of LevS. Joint participation of the N- and C-terminal domains is essential for stability, activity, specificity, and polymerization processes. Specifically, the N-terminal region is involved in stability, while the transition region plays an essential role in the transfructosylation reaction and polymer elongation. Based on our results, we suggest that ADs interact with each other, adopting a U-shaped topology. The importance of these ADs observed in the MD-FN of the Leuconostocaceae family is not shared by the Lactobacillaceae family. Phylogenetic analysis of LevS AD suggests that MD-FN from Lactobacillaceae and Leuconostocaceae have different evolutionary origins. This is the first study on the structure-function relationship of multidomain levansucrases from the Leuconostocaceae family. Our results point towards the functional role of AD in MD-FN and its involvement in fructan synthesis.

## 1. Introduction

Fructansucrases (FNs) are enzymes that are responsible for the synthesis of fructan, a fructose polymer with important applications in the food, cosmetic, and pharmaceutical industries [1,2,3,4,5,6]. Due to the wide range of fructan applications, the study of FNs has gained considerable interest. FNs catalyze the transference of the fructosyl moiety from sucrose to either a sucrose molecule or to a growing fructan polymer chain. This type of reaction is denominated transfructosylation. If the fructosyl moiety is transferred to a water molecule instead, hydrolysis of the glycosidic bond in sucrose occurs [7,8]. Levansucrases (E.C. 2.4.1.10) and inulosucrases (E.C. 2.4.1.9) are FNs which produce different types of fructans. Levansucrases (LSs) produce levan, a polymer containing fructose monomers linked by β (2-6) bonds. Inulosucrases (ISs) synthesize inulin, a polymer of fructose with β (2-1) bonds. The structural characteristics of these enzymes determine their regiospecificity and transference/hydrolysis specificity, as well as fructan size and branching [7,9].

FNs belong to the family 68 of glycosyl hydrolases, whose members display a catalytic domain with a five-bladed β-propeller architecture. Each β-sheet adopts a “W” topology with four antiparallel β-strands. The β-propeller contains a negatively charged funnel-like structure in the center. This structure has two aspartic acid residues acting as the nucleophile and transition state stabilizer, and one glutamic acid serves as a general acid/base residue [10]. The transfructosylation reaction is carried out in the presence of sucrose. FN catalyzes the transfer of the fructosyl moiety from sucrose to an acceptor molecule. In this enzymatic reaction, the sucrose glycosidic linkage is broken, resulting in the formation of a covalent fructosyl–enzyme intermediate and the release of glucose. Finally, the fructosyl residue is transferred to the acceptor molecule [11].

The complete levan synthesis pathway has been recently identified, pinpointing the amino acids that make up the five product/acceptor binding subsites. These subsites help the enzyme carry out fructan elongation [9]. FNs can synthesize polymers with different molecular weights by means of two distinct elongation mechanisms. The synthesis of low-molecular weight fructan employs a non-processive mechanism, where the fructose units are added to growing chains but are taken up and released into the solution according to their affinity for the enzyme. In contrast, the high-molecular weight fructan is produced through a processive mechanism, which consists of successive incorporations of fructose units into a single growing chain, retained by the enzyme until the elongation process is finished [12,13].

Most FNs consist of a single catalytic domain with an approximate molecular mass of 45 to 65 kDa. However, a cluster of FNs from Gram-positive bacteria, specifically from the Lactobacillales order, have a multidomain structure. Consequently, their molecular mass ranges from 80 kDa to 170 kDa. Multidomain FNs (MD-FNs) are composed of three regions: (I) an N-terminal domain, (II) a conserved catalytic domain, and (III) a C-terminal domain. The MD-FN architecture resembles the structure observed in glucansucrases (GNs), which are enzymes that belong to family 70 of glycosyl hydrolases, which also have a multidomain structure. These enzymes use sucrose as a substrate to synthesize a glucose polymer, denominated glucan [14]. Several groups have reported the identification and characterization of multi-domains in both LSs and ISs (MD-LSs and MD-ISs) (Table 1). However, the characterization of these enzymes has been carried using truncated versions that lack a certain domain. Furthermore, many of these enzymes have been predicted by genomic studies using annotations in GenBank, which is why we expect that the study of these enzymes will increase in the coming years.

To date, the only crystallographic structure described is that of MD-IS InuJ, a truncated version from *Lactobacillus johnsonii*. The structure of this version contains the catalytic domain and a minimal region containing the additional domains. The catalytic domain displays a typical β-propeller structure, with the catalytic site in the center and bottom of the funnel-like structure. Thirty-three residues of the N-terminal region were identified in the structure, which make up two motifs, α1 and α2 helices. The N-terminal domain extends away from the catalytic domain and is connected to it through 12 residues. Based on this structure, the author suggests that the N-terminal and C-terminal domains of InuJ are far from the active-site pocket. Therefore, it is unlikely that they are critical for the transfructosylation process [25].

Few researchers have addressed the structure–function relationship of additional domains in MD-FNs, probably due to the difficulty of producing and stabilizing these enzymes and their truncated versions. Moreover, the results of the limited studies regarding lactobacillus FN’s structure-function relationship are contradictory. Studies regarding fructan production employing truncated lactobacillus FN versions have identified that the additional domains are excluded from the catalytic process, suggesting that their participation is limited to the anchorage of FNs to the membrane of Lactobacillales, or to a lesser extent, to the stability of the protein structure [26,27]. On the other hand, a study of MD-ISs from a Leuconostocaceae family member reported an important participation of the additional domains for catalysis, highlighting their importance for enzyme stability and in the transfructosylation reaction [28].

Further studies are required to better understand the role of additional domains in the enzymatic activity of MD-FNs in Lactobacillales. Some studies have been performed on MD-FNs from the Lactobacillaceae family; however, information is lacking to fully understand the structure-function relationship of these ADs present in the Leuconostocaceae family. This study reports, for the first time, the role of these additional domains in the activity, stability, reaction specificity, and elongation mechanism of MD-LSs in the Leuconostocaceae family, using the LevS levansucrase from *Leuconostoc mesenteroides* B-512 F as a model. The present results, in conjunction with those obtained in studies performed using MD-ISs from *L. citreum*, will contribute to a better understanding of the catalysis reaction carried by MD-FNs from the Lactobacillales order.

## 2. Materials and Methods

### 2.1. Construction of LevS and Truncated Versions

The mature form of LevS and five different truncated versions were generated using the expression vector pBAD/Directional-TOPO ThioFusion (Invitrogen, Carlsbad, CA, USA) under the control of the L-arabinose promoter. It contains the His patch at the C-terminus and the thioredoxin leader at the N-terminus. LevS and LevSΔN were generated by inverse PCR using the pBAD-LevS construct generated by Morales-Arrieta as a template [17]. LevSΔC and LevSΔNC were generated using the truncated construction pBAD-LevSΔC as a template, which was derived from pBAD-LevS, but lacking the C-terminal domain of LevS. LevSΔTnC was generated using the truncated construction pBAD-LevSΔTnC as a template, which was derived from pBAD-LevS, but lacking the C-terminal domain and the transition region of LevS. LevS/Cat was generated using the truncated construction pBAD-LevS/Cat as a template, which was derived from pBAD containing only the catalytic domain of LevS. PCR was carried out using Vent DNA polymerase (NEB) to generate fragments flanked by the NcoI restriction sequence, as shown in Figure S1. Table S1 shows the primers used in PCR. The thioredoxin fusion protein from the N-terminus was also removed from the template. PCR products were digested with NcoI and ligated. Ligation products were transformed into *Escherichia coli* DH5α cells to propagate the plasmid constructions and cultured at 37 °C and 250 rpm in LB medium (Luria-Bertani) supplemented with 50 µg/mL kanamycin to maintain the integrity of the plasmid. Agar plates were made by adding 1.5% agar to LB medium. *E. coli* plasmid DNA was isolated using the Zippy Plasmid MiniPrep Plasmid Isolation Kit (Zymo Research). Restriction enzymes were purchased from Thermo Fisher Scientific (Waltham, MA, USA). In all cases, the biological reagents were used as recommended by the manufacturer.

### 2.2. Protein Expression of LevS and Truncated Versions

*E. coli* one-shot Top10 cells were used for enzyme production purposes. Overnight cultures carrying the appropriate LevS constructions and truncated versions were inoculated in LB medium with 200 µg/mL ampicillin. Cultures were grown at 37 °C and shaken at 200 rpm until OD_600 nm_ 0.5–0.6 was reached. Protein expression was induced by adding 0.02% (*w*/*v*) L-arabinose, incubating for an additional 4 h at 18 °C, and shaking at 120 rpm. Cells were harvested by centrifugation (10 min, 4 °C, 5000 g). The resulting pellet was washed twice with 50 mM sodium acetate buffer, pH 6. Cells were resuspended in lysis solution (1 mg/mL lysozyme) and frozen and thawed three times before sonication. Cell debris was removed by centrifugation (40 min, 4 °C, 10,000 g).

### 2.3. Activity Assay

Enzymatic activity was measured by detecting the reducing power released from 100 g/L of sucrose in 50 mM sodium acetate buffer (pH 6) with 1 mM CaCl_2_ at 30 °C, using the 3,5-dinitro-salicylic acid method. One unit of enzyme activity was defined as the amount of enzymes that catalyzes the production of 1 µmol of sugar reducing/min. This corresponds to a global LS activity, which includes transfructosylation and hydrolysis activities.

### 2.4. Analysis of the Synthesized Monosaccharide, Oligosaccharide, and Polymer Profile

Levan molecular weights were determined by high-performance size exclusion chromatography (HPSEC) by means of two SEC Ultrahydrogel columns in series (Ultrahydrogel Linear, 7.8 × 300 mm, and Ultrahydrogel 500, 7.8 × 300 mm) using 0.1 mM NaNO_3_ as eluent at a flow of 0.8 mL/min. Commercial 5.2–668 kDa dextrans (Sigma-Aldrich, St. Louis, MO, USA) were used as calibration standards. Transfructosylase and hydrolysis activities, as well as sucrose conversion, were determined from the concentrations of synthesized fructose, glucose, and sucrose. Fructose is a product of hydrolytic activity, whereas the difference between glucose and fructose concentrations is a result of transfructosylation activity. Simple sugar concentrations were analyzed by High Performance Anion-Exchange Chromatography with Pulsed Amperometric Detection (HPAEC-PAD) using a 4 × 250 mm Dionex Carbopac (Thermo Scientific, Waltham, MA, USA) PA1 column. An isocratic flow of 200 mM NaOH was applied at a flow rate of 0.5 mL/min and 30 °C. Oligosaccharide analysis was performed by HPAEC-PAD using a 4 × 250 mm Dionex Carbopac PA200 column. A gradient of sodium acetate (5–100 mM in 20 min, 100–400 mM in 60 and 10 min for initial conditions re-equilibration) with 150 mM NaOH at a flow rate of 0.5 mL/min was applied. Detection was performed using the Dionex ICS-5000 (Thermo Scientific, Waltham, MA, USA) module with a gold working electrode and a pH reference Ag/AgCl.

### 2.5. Polymer Synthesis

Polymer was produced with 1 U/mL of enzyme extract at 30 °C in 50 mM sodium acetate buffer (pH 6) containing 100 g/L sucrose and 1 mM CaCl_2_. Polymer precipitation was carried out with two volumes of ethanol, dialyzed against 10 mM sodium acetate buffer pH 6, lyophilized, and analyzed by ^13^C NMR.

## 3. Results

### 3.1. Construction of the LevS and Its Truncated Versions

**Figure 1:**
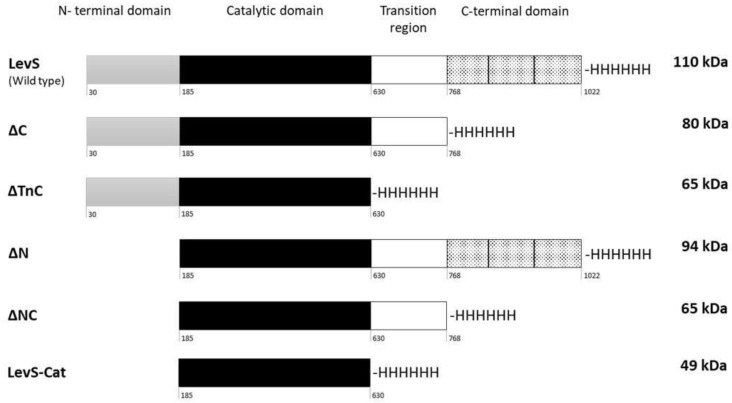
Schematic representation of LevS and truncated forms. LevS: complete mature enzyme; LevSΔC: deletion of the C-terminal domain; LevSΔTnC: deletion of the transition region and the C-terminal domain; LevSΔN: deletion of the N-terminal domain; LevSΔNC: deletion of both N- and C-terminal domains; LevS-Cat: presence only of the catalytic domain. All proteins contain a histidine tag at the C-terminus.

The MD-LS LevS is an extracellular enzyme containing 1022 amino acids (113 kDa), harboring three regions that have the structural characteristics of glucansucrases and fructansucrases [17]. The N-terminal region of LevS is made up of 185 amino acids. It includes the signal peptide (residues 1–30) and a region known in GNs as the variable region (residues 31–185). This domain is followed by the catalytic domain, consisting of 445 amino acids. Finally, there is a C-terminus, which is divided into a transition region (Tn) of 138 amino acids (residues 630–768) and a C-terminal domain of 254 amino acids (residues 769–1022) (Figure 1). For the heterologous expression of the complete LevS enzyme, we cloned a fragment that codes the mature LevS without fused proteins or signal peptide (100 kDa).

The functional role of the N- and C-terminal domains, as well as the Tn region, was studied by analyzing the biochemical properties and the reaction products of five truncated versions of LevS (Figure 1). LevSΔC was obtained through the deletion of 254 amino acids corresponding to the C-terminal domain. LevSΔTnC lacks the transition region and the C-terminal domain. LevSΔN corresponds to the deletion of the N-terminal domain. LevSΔNC corresponds to expression of only the catalytic domain and the transition region. Finally, LevS/Cat corresponds to the expression only of the catalytic domain. Proteolysis is a common phenomenon observed in GTFs of *Leuconostoc* spp. expressed in *E. coli*, which does not decrease, despite the presence of protease inhibitors [29]. SDS-PAGE gels showed that the non-degraded recombinant proteins are predominant in fresh crude enzyme extracts (data not shown). Furthermore, *E. coli* is unable to produce FNs naturally; therefore, fresh extracts were used for biochemical characterization.

### 3.2. Biochemical Characterization and Stability of LevS from L. mesenteroides NRRL B512F and Its Truncated Versions

To determine the effect of deleting certain domains on the biochemical properties of the enzymes, we evaluated optimal pH, temperature, and stability of the complete LevS and its truncated versions (Table 2). LevS has an optimal pH of 6, as reported by Morales-Arrieta [17]. Deletion of the C-terminal domain both alone and in conjunction with the transition region decreased the optimal pH, unlike the removal of the N-terminal domain, which did not affect this parameter. In contrast, the value of this parameter increased when both terminal domains were removed and when only the catalytic domain remained. These results show the importance of both the N- and C-terminal domains in maintaining the optimal pH.

The optimal temperature for the truncated versions was evaluated at pH 6.0. The optimal temperature of LevS was 35 °C, similar to that previously reported [17]. The elimination of the C-terminal domain did not affect the optimal temperature of LevSΔC; however, removal of the C-terminal domain and the Tn region (LevSΔTnC) decreased the optimal temperature by 5 °C. Interestingly, a greater change was observed when eliminating the N-terminal domain (LevSΔN). In this case, the optimal temperature decreased by 10 °C. This effect was maintained when both the N- and C-terminal domains were deleted (LevSΔNC). Finally, when all additional regions were removed (LevS-Cat), the catalytic domain returned to the optimal temperature as in LevS. These results suggest that the N-terminal domain and the Tn region are implicated in the optimum temperature, probably through stabilization of the enzyme.

To demonstrate this hypothesis, we analyzed the stability of the truncated versions. Stability was measured as the residual activity after incubating the enzyme extract at 30 °C for 24 h. The C-terminal domain and the transition region had no influence on the stability of LevS since there was no change in the activity of LevSΔC and LevSΔTnC. However, the N-terminal domain directly affects the stability of LevS, as the activity of LevSΔN decreased to 47%. The activity of LevSΔNC and LevS-Cat decreased to an extent that it could not be measured. Therefore, the N-terminal domain seems to be strongly involved in the stability of the enzyme; however, the presence of the N- and C-terminal domains is essential to maintain LevS enzymatic property. Despite its low activity, LevSΔNC and LevS/Cat were able to convert 20% of the sucrose after 24 h of reaction, which was enough to analyze their specificity and the reaction products.

### 3.3. Effect of the Deletion of Additional Domains of LevS on the Reaction Rate and H/T Specificity

The catalytic rate was analyzed by measuring the free reducing sugars released during the reaction (sucrose consumption). This analysis includes both transfructosylation and hydrolysis activities, determined at 30 °C, pH 6, and 100 g/L of sucrose. Most versions reached 80% of sucrose consumption (data not shown).

**Figure 2:**
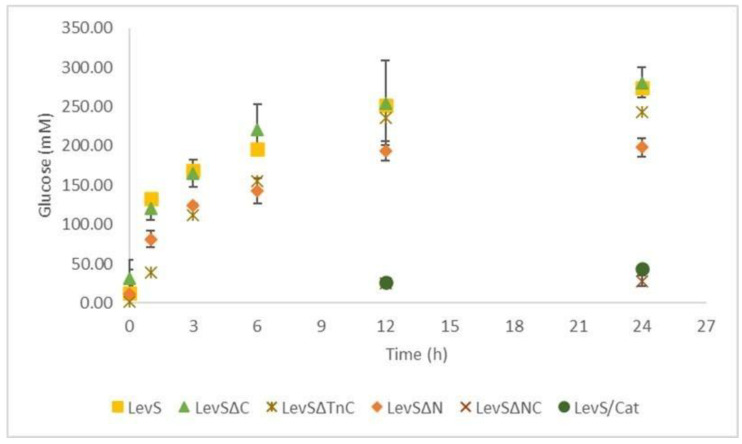
Effect of the additional domains of LevS on the enzymatic reaction. Reaction progress on 100 g/L sucrose and 1 U/mL enzyme for LevS and truncated versions.

LevS and LevSΔC have a similar catalytic rate, whereas this rate is slightly decreased in LevSΔN and LevSΔTnC. This reduction was more drastic in LevSΔNC and LevS/Cat. The decrease in the catalytic rate of some truncated versions impacted its efficiency of substrate consumption at the end of the reaction (24 h). This is the case of LevSΔN, which showed reduced efficiency by approximately 25% when compared to LevS, while LevSΔNC and LevS/Cat showed a decreased efficiency of 90% (Figure 2). These results could indicate that the N- and C-terminal domains jointly participate in the catalytic rate and the efficiency of substrate consumption.

**Figure 3:**
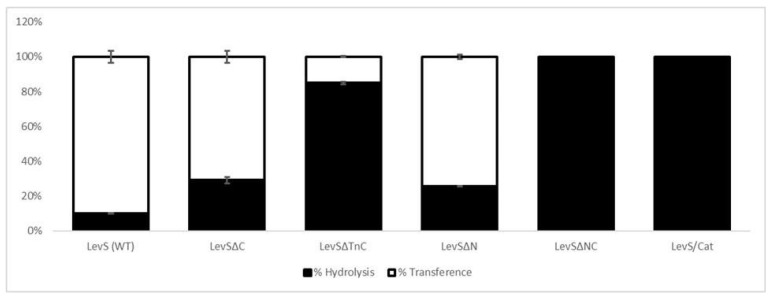
Hydrolysis and transfructosylation specificity of LevS and truncated forms. Reactions were carried out at 30 °C in 50 mM acetate buffer (pH 6) containing 1 mM CaCl2 and 290 mM sucrose as substrate.

The predominant transfructosylase activity of LevS was moderately disturbed when any of the terminal domains were missing, as this function decreased by 17% and 21% in the LevSΔC and LevSΔN variants, respectively. On the other hand, the enzyme activity became predominantly hydrolytic in LevSΔTnC, showing the importance of both of the missing regions in the transfructosylation reaction. Furthermore, the presence of the Tn region in LevSΔNC was not enough to maintain the transfructosylase activity of the enzyme, rendering it a 100% hydrolytic enzyme, similar to the LevS/Cat version. These results suggest that the Tn region participates in conjunction with the N- or C-terminal domains so that the catalytic domain develops this transfructosylase activity (Figure 3).

### 3.4. Profile of Products Synthesized by LevS and Its Truncated Versions

**Figure 4:**
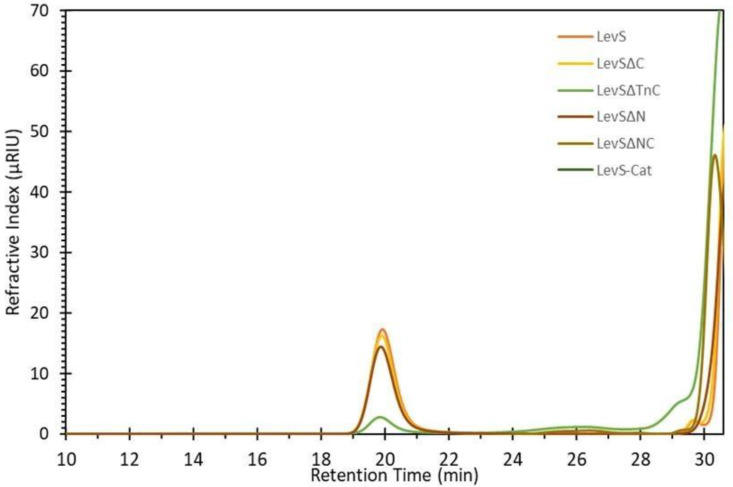
HMW polymer profile obtained from sucrose by LevS and truncated versions. Reaction conditions: enzyme 1.0 U/mL, sucrose 292 mM, pH 6, 30 °C.

Figure 4 shows the chromatograms of the products synthesized by all the truncated versions at 90% of sucrose conversion, except for LevSΔNC and LevS/Cat, which achieved only 20% conversion. LevS mainly produces a levan-type high-molecular weight (HMW) polymer with an approximate molecular weight of 2200 kDa, as reported by Morales-Arrieta [17]. The HMW polymer synthesis decreased by 6.6% in LevSΔC when compared to the WT but was drastically reduced (92.2%) in the LevSΔTnC version, which also yielded smaller synthesis products than those of WT and LevSΔC. These observations highlight the importance of the Tn region in the polymer elongation process developed by LevS. On the other hand, HMW polymer synthesis was slightly decreased in LevSΔN, whereas LevSΔNC and LevS/Cat did not show HMW polymer synthesis capacity (Figure 4).

**Figure 5:**
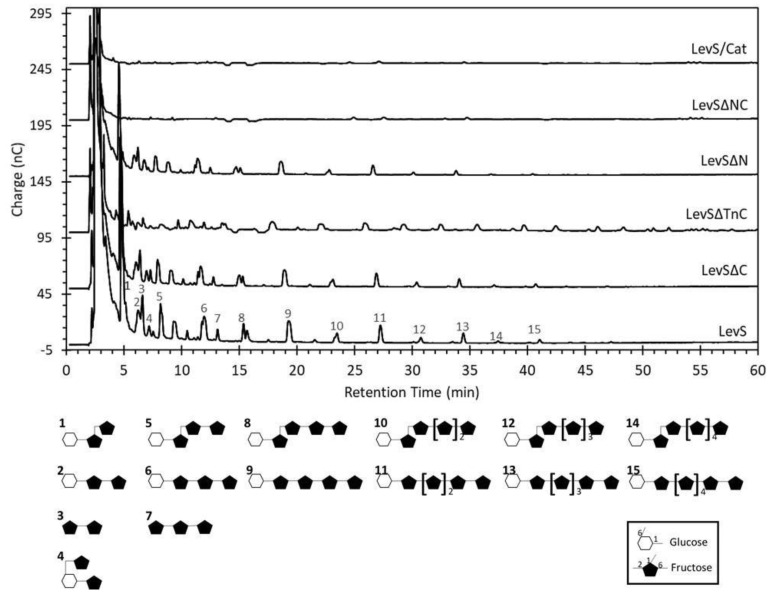
Oligosaccharide profile synthesized by LevS and truncated versions measured by HPAED-PAD. Reaction conditions: 1.0 U/mL enzyme, 292 mM sucrose, 30 °C, and pH 6. Reactions were analyzed at a sucrose conversion over 90%, except LevSΔNC and LevS/Cat, which reached 20%. Structures included are 1-kestose (1), 6-kestose (2), levanbiose (3), neo-kestose (4), 1,6-nystose (5), 6,6-nystose (6), levantriose (7), 1,6,6-kestopentaose (8), 6,6,6-kestopentaose (9), 1,6,6,6-kestohexaose (10), 6,6,6,6-kestohexaose (11), 1,6,6,6,6-kestoheptaose (12), 6,6,6,6,6-kestoheptaose (13), 1,6,6,6,6,6-kestooctaose (14), and 6,6,6,6,6,6-kestooctaose (15).

A detailed analysis by HPEAC-PAD of the products of LevS and its truncated versions showed that LevS additionally synthesizes low-molecular weight (LMW) products (Figure 5). The comparison with standards allowed the identification of these products: the first and most intense signals eluting before 4 min correspond to glucose, fructose, and residual sucrose, followed by the disaccharide levanbiose [D-Fru*f*-(2→6)-β-D-Fru*f*], the trisaccharides 1-kestose [β-D-Fru*f*-(2→1)-β-D-Fru*f*-(2→1)-α-D-Glc*p*], 6-kestose [β-D-Fru*f*-(2→6)-β-D-Fru*f*-(2→1)-α-D-Glc*p*], and neo-kestose [β-D-Fru*f*-(2→6)-α-D-Glc*p*-(1→2)-β-D-Fru*f*] as transfer products to sucrose, and levantriose [β-D-Fru*f*-(2→6)-β-D-Fru*f*-(2→6)-β-D-Fru*f*], a trisaccharide of the oligolevan series. Additionally, two series of levan-type fructooligosaccharides (FOS) were identified by comparison with standards: one derived from the elongation of 1-kestose (1K-FOS, including products 1, 5, 8, 10, 12, and 14 in Figure 5), and the second derived from 6-kestose (6K-FOS, products 2, 6, 9, 11, 13, and 15 in Figure 5). The latter series showed greater intensity, but both reached a degree of polymerization (DP) of 8. Other products were also observed; however, they could not be identified as they did not match the standards.

The LMW product profile synthesized by LevSΔN and LevSΔC remained un-changed. In contrast, for LevSΔTnC, the production of 1K-FOS and 6K-FOS series was detected up to approximately DP10, corroborating the effect on the elongation capacity of the enzyme observed in HPSEC analysis. Finally, for LevSΔNC and LevS/Cat enzymes, no LMW products were observed, supporting the observation that sucrose was only hydrolyzed (Figure 5).

By comparing the distribution of fructose in the reaction products, it was possible to quantify the hydrolytic activity and the effect on the transferase activity to high- and low-molecular weight products between the different versions of LevS. As shown in Table 3, the primary transfer product of LevS was the HMW polymer and, to a lesser extent, the LMW products, and the hydrolytic activity represented just 10% of the total activity. The LevSΔC enzyme became more hydrolytic (29.1%) but maintained a profile similar to LevS; the HMW polymer was the main synthesis product (68.9%). We observed a change in the product profile for the LevSΔTnC enzyme since hydrolysis became the main activity (85%). Furthermore, the amount of HMW polymer synthesized drastically decreased by 92.2%, and the synthesis of LMW products slightly decreased by 42.7%. The hydrolytic activity, as well as the product profile of LevSΔN, was similar to LevSΔC, producing mainly HMW polymers and, to a lesser extent, LMW products. The product profile of LevSΔNC and LevS/Cat was evaluated at 20% sucrose conversion, where the main activity of the enzymes was hydrolysis. These results show the importance of the LevS additional domains, especially the Tn region, in the elongation of the HMW polymer.

## 4. Discussion

### 4.1. Role of Additional Domains on the Biochemical Properties and Stability of LevS

Proteins are stabilized by different intermolecular forces: hydrogen bonds, hydrophobic interactions, disulfide bonds, charge–charge interactions, salt bridges, and π* interactions. Subtle changes in the displacement of the atoms due to amino acid deletion change the conformation of the protein, increasing or decreasing its stability. Thus, in the case of an enzyme, the elimination of several amino acids can change the optimum conditions of reaction such as stability, leading to enzyme denaturation [30].

Our results showed that the truncated versions of LevS displayed modified biochemical properties. In the case of optimal pH, both the N- and C-terminal domains were implicated in maintaining the optimal pH of 6. It is important to highlight that the role of the additional domains has not been studied in other MD-LV from *Leuconostoc*, and only a few studies have addressed the AD impact in MD-LV from Lactobacillales. Van Hijum et al. (2004, 2008) and Ni et al. (2021) performed and characterized C- and N-terminal domain truncated versions of MD-IN and MD-LV from *Lactobacillus reuteri*; however, they did not compare the effects with the WT versions (both MD-IS and MD-LV) [26,31,32]. Del Moral et al. (2008) did not report changes in the optimal pH between WT MD-IS IslA and truncated versions in the N- and C-terminal domains [28]. Similarly, Pijning et al. (2011) did not observe changes in the optimal pH of the MD-IS InuJ of *Lactobacillus johnsonii* [25].

Regarding optimal temperature, the C-terminal domain does not seem to be related to this property; however, both the N-terminal domain and the Tn region are involved in the optimum biocatalysis temperature. In a similar way, Del Moral et al. (2008) observed that the optimum temperature (35 °C) was unchanged when eliminating the C-terminal domain from the MD-IS IslA from *Leuconostoc citreum* CW28. Nonetheless, removal of the C-terminal domain and the Tn region caused a decrease in the optimal temperature by 5 °C in the truncated version IslA3. This change was maintained in the truncated version IslA4, which maintains only the catalytic domain and 100 amino acids from the N-terminal [28]. In the *Lactobacillus johnsonii* NCC 533 MD-IS InuJ, the truncated versions did not show optimal temperature changes (55 °C) [33].

To demonstrate if the observed changes in the biochemical properties were related to enzymatic stability, we analyzed this parameter. Our results indicate that the N- and C-terminal domains are jointly essential for enzyme stability, but not independently. These results resemble those reported for the MD-IS IslA of *L. citreum*, where it was observed that the N- and C-terminal domains contribute to the stabilization of the catalytic domain, since the elimination of both domains resulted in a threefold decrease in stability compared to the WT enzyme [15,28]. However, enzyme stability was unaffected by removal of the C-terminal domain and the Tn region. This effect in stability is similar to that reported for the DsrS glucansucrase from *Leuconostoc mesenteroides* NRRL B-512F, where truncated versions in the C-terminal domain were found to be more susceptible to thermal denaturation [34]. Recently, Ni et al. (2021) observed a decrease in the stability of the N-terminal truncated variants of MD-IS and LS from *L. reuteri* 121, in accordance with those observed in the MD-IS IslA [26].

In general, we can conclude that the N- and C-terminal domains are involved in the enzymatic stability of LevS, probably through intermolecular forces from the amino acids that compose them, with the N-terminal having a more relevant role. A destabilization of the enzyme folding is perhaps responsible for the changes in biochemical properties in the truncated versions.

### 4.2. Function of the Additional Domains in the Reaction Rate and Polymer Elongation

It has been demonstrated that the enzyme nature and the reaction conditions are important aspects of fructansucrase catalysis [13]. The MD-FNs from the *Leuconostococacea* family, which are more efficient for transfructosylation reactions, have a more complex structure than single-domain enzymes [28]. In this work, we found that deletion of the N-terminal domain generates an enzyme with a reduced catalytic rate and is consequently less efficient in the substrate consumption (25% at 24 h); however, the joint elimination of the N- and C-terminal domains, including the Tn region or not, practically eliminated the catalytic rate, affecting the final efficiency of the enzyme by 90%. Our observations suggest that N- and C-terminal domains participate jointly in the overall activity of the enzyme (Figure 2), probably favoring the stability of the enzyme.

Regarding reaction specificity, although LevS has a predominant transfructosylase activity, the enzyme became mainly hydrolytic when one of the terminal domains was missing, including the Tn region (Figure 3). These results demonstrate that the participation of both N- and C-terminal domains is necessary to efficiently develop the transfructosylation reaction. Consequently, this effect could also be related to the stability of the enzyme, and the experiment probably only showed the early stages of the reaction. However, if we compare the LevS hydrolysis/transference ratio (H/T ratio) with the truncated version that lacks N- and C-terminal domains at 20% conversion, we observed that the H/T ratio is not similar. In fact, while the LevS enzyme has 90% transferase activity, the truncated version is fully hydrolytic, suggesting that observations at 20% sucrose conversion are sufficient to support the involvement of the N- and C-terminal domains in the transfructosylation reaction.

Interestingly, the strong difference observed in transfructosylase activity between the LevSΔC and LevSΔTnC versions, both C-domain-lacking enzymes with and without the Tn region, respectively, highlights the importance of the Tn region in the reaction specificity. In this case, stability was not compromised. Therefore, the Tn region has an important role in the enzymatic catalysis. Currently, these regions have not been studied in other MD-FNs.

Two mechanisms of polymer elongation have been reported for FNs. The processive mechanism is characterized by a low synthesis of FOS with low DP, and the predominant synthesis of HMW polymers. In contrast, the non-processive mechanism involves the formation of oligosaccharides that gradually grow and form a distribution of LMW products [12]. Accordingly, we can conclude that LevS synthesizes HMW polymer by a processive mechanism, as the accumulation of FOS with DP < 10 is scarce. The low FOS synthesis and the HMW polymer presence support this affirmation. This profile is similar to that reported for the levansucrase LevG and the inulosucrases (InuGA, InuGB) from *Lactobacillus gasseri* [24].

Removal of both the N- and C-terminal domains in the truncated version LevSΔNC drastically affected HMW polymer synthesis capacity, as well as the elimination of all the additional domains in the truncated LevS/Cat version (Figure 4). In contrast with these results, for IslA MD-IS from *L. citreum*, no change was observed in the distribution of the HMW polymer produced by IslA and its truncated versions in the N- and C-terminal domains [28]. In enzymes from the Lactobacillaceae family, the effect of the N-terminal domain on the synthesis capacity of HMW products was also observed in the MD-LS from *Lactobacillus reuteri* 121; however, in the MD-IS of this same microorganism, the elimination of this region results in some minor changes to the product profiles [26]. Similarly, the C-terminal truncated version of MD-LS from *L. reuteri* also did not show any changes in the distribution of synthesized HMW polymers [31].

The LevS enzyme produces FOS up to DP8; however, the truncated versions LevSΔNC and LevS/Cat are hydrolytic since no LMW products were observed, showing that the N- and C-terminal domains are necessary for the transfructosylation reaction. These results contrast with those reported for the N- and C-terminal domain truncated version of the inulosucrase InuJ from *L. johnsonii*, which synthesizes FOS from inulin with a DP15, while the WT enzyme synthesizes only kestose and HMW polymers [33]. On the other hand, the truncated version in the N-terminal domain of levansucrase from *L. sanfranciscensis* TMW 1.392 did not show a change in the FOS distribution concerning the WT enzyme [22].

We estimated the specificity of the transfructosylation reaction to produce HMW polymers or LMW products for LevS and the different truncated versions (Figure 3). We observed that the Tn region is crucial for the transfructosylation reaction for HMW polymer synthesis, and thus to define the elongation mechanism. However, the N- or C-terminal domains complement the Tn region, making MD-LV more efficient. The evidence from this study points towards the idea that the additional domains are implicated in the enzyme activity, participating in a meaningful manner in the transfructosylation reaction and in the polymer elongation, allowing MD-FNs to synthesize FOS and HMW polymers.

**Figure 6:**
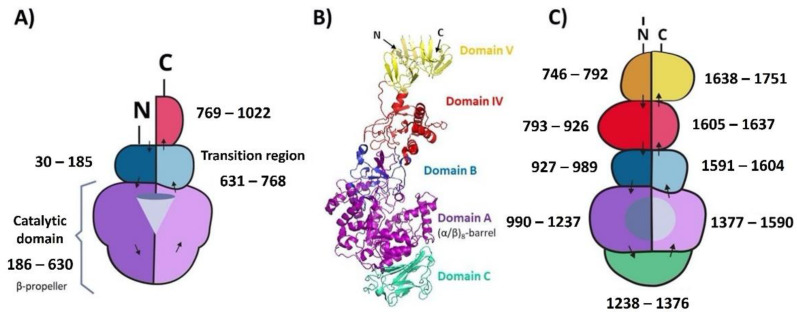
Hypothetical schematic representation of the LevS levansucrase from *Leuconostoc mesenteroides*. (**A**) LevS could have a U-shaped folding similar to that of glucansucrases. (**B**) Crystal structure of GTF180-ΔN. (**C**) Schematic representation of the glucansucrase GTF. Modified from Vujičić-Žagar et al. (2010) [36].

Our results support the idea that LevS ADs are associated and interact with the catalytic domain. Particularly, the N-terminal and Tn region could interact jointly with the catalytic site and participate essentially in the transference of the fructosyl unit to the acceptor molecule. This role resembles those observed in the additional domains present in the glucansucrases of the GH 70 family. These multidomain enzymes have a U-shaped fold where the additional N- and C-terminal domains interact with each other, giving rise to five structural domains. Domain A is the catalytic domain, domain B contributes several amino acid residues to the substrate acceptor and donor binding sites, and domain C is the base of the “U”. Domain IV functions as a hinge, helping domain V to bring the acceptor closer to the catalytic site [35]. Therefore, we propose that LevS levansucrase could have a similar folding, where the additional N- and C-terminal domains and the transition region are associated (Figure 6). We hypothesize that the catalytic domain of LevS would comprise the A and C domains of a glucansucrases, while the B domain would comprise the transition region and the N-terminal domain. Finally, the C-terminal domain of LevS would act as a hinge that supports the approach of the growing polymer to the catalytic site. This arrangement favors the activity of LevS, making it a more efficient transferase.

### 4.3. Comparison of Additional Domains Role in MD-FNs from the Leuconostocaceae and Lactobacillaceae Families

As we mentioned previously, very few researchers have studies the structure–function relationship of additional domains in MD-FNs of the *Lactobacillales* order. For instance, in the case of MD-FNs from the Lactobacillaceae family, Van Hijum et al. (2002) studied a truncated version of MD-IN from *Lactobacillus reuteri,* which lacks the C-terminal region implicated in anchoring the enzyme to the cell wall. The authors found no difference in the reaction products synthesized by the C-terminal truncated version compared to those produced by purified enzymes from the wild-type strain [27]. On the other hand, Tieking et al. (2005) analyzed a truncated version of MD-LS from *Lactobacillus sanfranciscensis* that lacks the N-terminal repeats. They found no effect on the kinetic properties of the MD-LS or the product profile generated from sucrose when removing the N-terminal repeating units [22,37]. Ni et al. (2021) recently generated and characterized truncated versions of the MD-IS and LS from several strains of Lactobacillales. These truncated enzymes displayed different effects on activity, highlighting the truncated IS and LS from *L. reuteri*, which showed the highest activity among all the truncated versions studied. The authors conclude that N-terminal truncation has little influence on fructan biosynthesis [26]. These reports suggest that the C- and N-terminal regions in MD-FNs from lactic acid bacteria have low contributions to catalytic activity and specificity [37].

In contrast, studies regarding the structure-function relationship of MD-IS from the *Leuconostocaceae* family have shown the importance of the N- and C-terminal regions in the catalytic capacity of these enzymes. Olivares-Illana et al. (2003) identified and characterized the MD-IS IslA from *Leuconostoc citreum* CW28. They also constructed two truncated versions lacking the C-terminal region and found a negative impact on transferase activity and enzyme stability [15]. Subsequently, Del Moral et al. (2008) conducted an in-depth study of the role of the additional domains in IslA, demonstrating that the C-terminal domain is involved in cell wall anchoring of the enzyme and establishing that both N- and C-terminal domains affect the substrate affinity (Km) and transfructosylation activity (H/T rate). The authors suggest that additional domains interact with the catalytic site, conferring rigidity to the enzyme and therefore stability [28]. Based on this hypothesis, four chimeras were designed and characterized attached to the SacB C-terminal domain and the transition region (Tn) of an MD-ISs (IslA) and a MD-LSs (LevC). The C-terminal and Tn bound to SacB changed the specificity of the SacB domain, achieving a fivefold increase in the transfructosylation catalytic constant (Kcat) without reducing the hydrolytic Kcat. The previous results suggest that the C-terminal domain and the Tn region modify the catalytic domain, producing a structural adjustment that increases affinity for the polymer acceptor molecule and the transferase efficiency [38]. Our analyses support the importance of the additional domains in the stability, specificity, and elongation mechanism of MD-FNs such as LevS from *L. mesenteroides*. Finally, our work supports the hypothesis that these additional domains have allowed these proteins to evolve towards enzymes with a better transfructosylation capacity.

The similar architecture of the MD-FNs from the Lactobacillaceae family suggests that these domains have a common evolutive origin. However, the difference in the functions of additional domains from MD-FNs in Lactobacillaceae and Leuconostocaceae families suggests that these domains diverged their evolutive function at some point in time. A phylogenetic analysis of the C-terminal and N-terminal additional domains for both the Leuconostocaceae and Lactobacillaceae families demonstrates that the additional domains from the Leuconostocaceae family belong to other clads, and its roots diverged recently in time from the AD belonging to MD-FNs from the Lactobacillaceae family (Figure S2). Based on these results, we can conclude that the additional domains diverged differently. Therefore, they have different functions than these MD-FNs from the Lactobacillaceae and Leuconostocaceae families.

## 5. Conclusions

In this work, we studied the role of the additional domains of MD levansucrase LevS from *Leuconostoc mesenteroides* B-512 F. Our analyses showed the participation of the additional domains in several biochemical properties and enzymatic catalysis. The N-terminal is relevant to stability; however, the conjunction of both N- and C-terminal domains is critical to the stability and, therefore, the enzymatic activity. This domain conjunction is also involved in specificity and the polymerization reaction. On the other hand, the Tn region is implicated in the transfructosylation reaction and polymer elongation, highlighting the importance of this region for catalysis developed by the LevS enzyme. The relevance of the conjunction of additional domains in catalysis leads us to suggest that the MD-FNs of the Leuconostocaceae family adopt a U-shaped topology where the additional domains interact with each other, similar to those described in the MD-GN enzymes. Finally, the importance of the additional domains observed in the FNs of the Leuconostocaceae family is not shared among MD-FNs of the Lactobacilleaceae family. Therefore, we suggest that although the architecture of the MD-FNs for the order of Lactobacillales is similar, the additional domains diverged evolutionarily, giving them different functions.

## Figures and Tables

**Table 1:** Multidomain fructansucrases from *Lactobacillales* order.

Microorganism	Enzyme	Mw (kDa)	GenBank Accession	References
*Leuconostoc citreum* CW28 (IslA)	IS	170 ^a^	AAO25086	[15]
*Leuconostoc mesenteroides* ATCC 8293	LS	ND	ABJ62504	[16]
*Leuconostoc mesenteroides* B512-F (LevS)	LS	130 ^a^	AAY19523	[17]
*Leuconostoc mesenteroides* B512-F (LevC)	LS	130 ^a^	ABJ62503	[18]
*Leuconostoc mesenteroides* B512-F (LevL)	LS	113 ^b^	ABJ62504	[18]
*Leuconostoc mesenteroides* Lm17	LS	120 ^a^	ALF07532	[19]
*Leuconostoc mesenteroides* LBAE-G15	LS	113 ^b^	AMD77912	[19]
*Leuconostoc mesenteroides* MTCC 10,508 (TrLmLevS)	LS	108 ^a^	QAU55073	[20]
*Lactobacillus reuteri* 121	LS	90 ^a^	AAO14618	[21]
*Lactobacillus sanfranciscensis* TMW 1.392	LS	105 ^a^	AEN98680	[22]
*Lactobacillus panis* TMW 1.648	LS	87 ^b^	WP_082611621	[23]
*Lactobacillus gasseri* DSM 20,077 (LevG)	LS	84 ^a^	ACZ67287	[24]
*Lactobacillus johnsonii* NCC 533 (InuJ)	IS	87.2 ^b^	AYN50318	[25]

ND, not described. ^a^ Experimental data based on SDS-PAGE analysis. ^b^ Calculated data according to the amino acid sequence.

**Table 2:** Biochemical characterization of LevS and truncated versions.

ENZYME	OPTIMAL TEMPERATURE (°C)	OPTIMAL pH	RESIDUAL ACTIVITY (%) **
LevS (WT)	35	6	96.97 ± 7.96
LevSΔC	35	5	100.00 ± 1.58
LevSΔTnC	30	5	98.90 ± 0.06
LevSΔN	25	6	47.85 ± 0.93
LevSΔNC	25	7	*
LevS-Cat	35	7	*

* It could not be determined due to the low enzyme activity in the extract. ** Incubation at 30 °C for 24 h.

**Table 3:** Percentage of fructose used for HMW polymer synthesis, LMW products synthesis, and hydrolysis.

ENZYME	HMW POLYMER	LMW PRODUCTS	HYDROLYSIS
LevS (WT)	73.8 ± 0.38	16.2 ± 3.38	10.0 ± 0.10
LevSΔC	68.9 ± 1.82	2.0 ± 0.33	29.1 ± 1.74
LevSΔTnC	5.7 ± 0.36	9.3 ± 1.20	85.0 ± 0.80
LevSΔN	73.1 ± 2.21	1.2 ± 0.32	25.7 ± 0.23
LevSΔNC *	0.0	0.0	100
LevS/Cat *	0.0	0.0	100

* Only reached 20% of sucrose conversion.

## Data Availability

Not applicable.

## Supplementary Materials

The following are available online at https://www.mdpi.com/article/10.3390/microorganisms10050889/s1, Figure S1: Strategy used for the elaboration of LevS and truncated version plasmids; Figure S2: Phylogenetic analysis of the additional domain from MD-FNs; Table S1: Primers used in this study. NcoI restriction site is underlined.
