# Supplementary material for: Structure–Function Relationship Studies of Multidomain Levansucrases from Leuconostocaceae Family
Source: Microorganisms. 2022 Apr 24;10(5):889. doi: 10.3390/microorganisms10050889 (PMC9142893; doi:10.3390/microorganisms10050889)
Supplement: Supplementary file 1 [file microorganisms-10-00889-s001.zip › microorganisms-1561497-supplementary.pdf]

Supplementary figures

Table S1. Primers used in this study. NcoI restriction site is underlined.

Primer	***Sequence (5' to 3')***
SPS-NcoI	CAT GCC ATG GAT ACT ACG AAC AGT ACA ACT
SNT-NcoI	CAT GCC ATG GGA AAA AAT GCT GAT GGT ACG
pBAD-NcoIRv	CAT GCC ATG GGT ATG TAT ATC TCC TTC TTA AAG TT

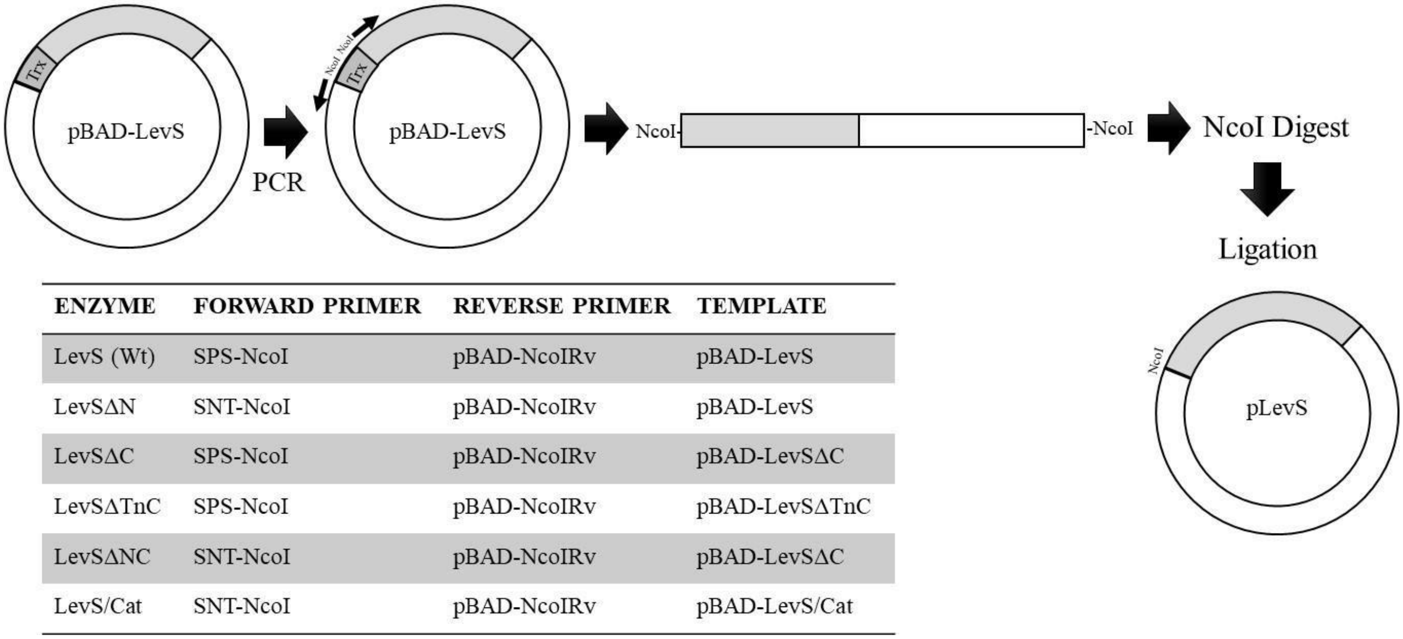

ENZYME	FORWARD PRIMER	REVERSE PRIMER	TEMPLATE
LevS (Wt)	SPS-NcoI	pBAD-NcoIRv	pBAD-LevS
LevSΔN	SNT-NcoI	pBAD-NcoIRv	pBAD-LevS
LevSΔC	SPS-NcoI	pBAD-NcoIRv	pBAD-LevSΔC
LevSΔTnC	SPS-NcoI	pBAD-NcoIRv	pBAD-LevSΔTnC
LevSΔNC	SNT-NcoI	pBAD-NcoIRv	pBAD-LevSΔC
LevS/Cat	SNT-NcoI	pBAD-NcoIRv	pBAD-LevS/Cat

Figure S1. Strategy used for the elaboration of LevS and truncated version plasmids. Table shows the primers and templates used for each enzyme.

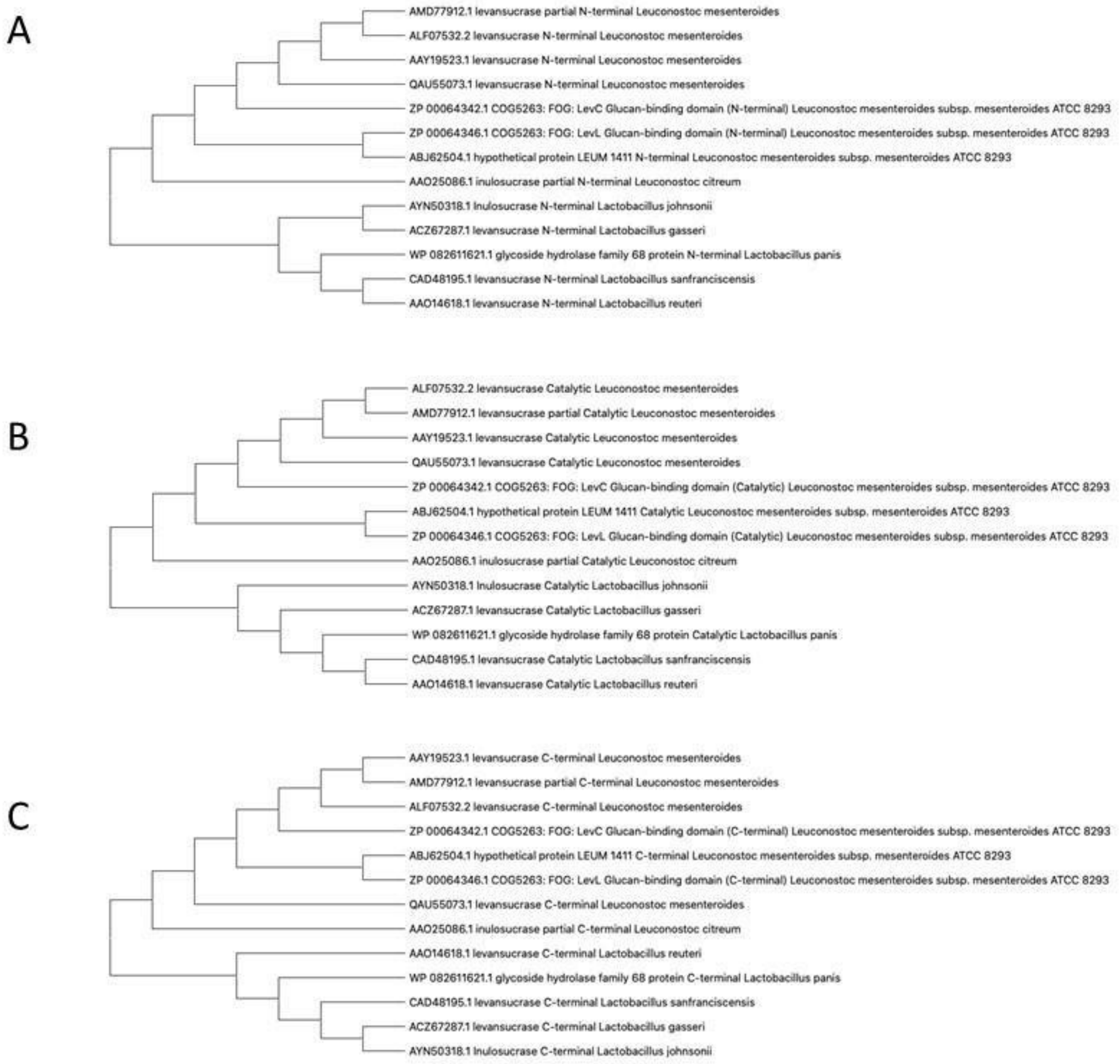

Figure S2. Phylogenetic analysis of the aditional domain from MDFNs. A) Phylogenetic analysis of the N-terminal region form MDFNs. B) Phylogenetic analysis of the MDFNs catalytic domain. C) Phylogenetic analysis of the C-terminal region form MDFNs.
